# Efficacy of wood ash and diatomaceous earth against *Sitophilus granarius*: influence of dose, environmental conditions, and geomorphological composition

**DOI:** 10.1093/jee/toaf109

**Published:** 2025-06-12

**Authors:** Miha Curk, Tanja Bohinc, Stanislav Trdan

**Affiliations:** Department of Agronomy, Biotechnical Faculty, University of Ljubljana, Ljubljana, Slovenia; Department of Agronomy, Biotechnical Faculty, University of Ljubljana, Ljubljana, Slovenia; Department of Agronomy, Biotechnical Faculty, University of Ljubljana, Ljubljana, Slovenia

**Keywords:** inert, dust, probit, progeny

## Abstract

This study evaluates the insecticidal efficacy of wood ash derived from various coniferous sources and a commercial diatomaceous earth product SilicoSec against adults of granary weevil, *Sitophilus granarius* (L.) (Coleoptera: Curculionidae). Morphological analyses revealed significant differences in particle sizes among treatments, with SilicoSec averaging 14.70 ± 1.85 µm, and wood ashes ranging from 160.51 ± 58.25 to 500.10 ± 183.58 µm. Geochemical analyses indicated that SilicoSec comprised 79.5% SiO_2_, whereas wood ashes contained 12.8% to 17.5% SiO_2_ and 31.0% to 37.6% CaO. Insecticidal assays demonstrated that all treatments achieved over 90% mortality; however, wood ash treatments exhibited greater variability, suggesting a dependence on concentration and environmental conditions. Probit modeling estimated that mortality increased by a factor of 2.77 with each concentration doubling. Analysis of variance indicated that neither temperature, nor relative humidity significantly affected lethal concentrations (LC_50_ and LC_90_). Notably, the diatomaceous earth product SilicoSec achieved consistent results at lower concentrations than currently recommended, suggesting potential for reduced application rates. Progeny emergence studies revealed that treatment efficacy was influenced by temperature and relative humidity, with higher temperatures and lower humidity levels enhancing mortality. Beta regression analysis confirmed that treatments and temperature significantly affected the proportion of dead beetles, whereas relative humidity and concentration did not. These findings underscore the importance of selecting appropriate treatment combinations and storage conditions to effectively control *S. granarius* populations.

## Introduction

The granary weevil, *Sitophilus granarius* (L.) (Coleoptera: Curculionidae) is a widely present insect pest responsible for significant economic losses in grain storage facilities worldwide. Wood ash has been proven in the past to be an effective natural replacement for conventional pesticides in many applications, including storage pest control (Jean et al. 2015, Bohinc and Trdan 2017, Milosavljević et al. 2024). It has been previously shown that wood ash from different tree species or geographical locations could have different insecticidal rates (Bohinc et al. 2018). Diatomaceous earth products are also known for their high effectiveness (Mortazavi et al. 2024) in that regard but are somewhat expensive and could be replaced by other locally abundant substances like wood ash if they prove sufficiently effective. However, despite both substances’ natural origin, concerns for their use arise, as they must be mixed with grain in storage facilities and could either contaminate milled products (Mortazavi et al. 2020), or as is the case with the naturally abrasive diatomaceous earths, cause excessive wear on the equipment (Lošić and Korunić 2017). Consequently, the right dosage of each substance is crucial to enable sufficient insecticidal properties but prevent unwanted consequences as well as reduce the costs of use.

Previous studies have shown that Probit analysis is promising for balancing tradeoffs between insecticidal properties and side-effects by determining expected values for lethal concentrations like LC_50_ or LC_90_, as was showcased in the past (Korunić et al. 2020). Shams et al. (2011) studied different diatomaceous earth dosages on several organisms, including *S. granarius*, but under constant environmental conditions. A similar study was conducted by Traian et al. (2015), where several local diatomaceous earth samples were compared to a commercial product SilicoSec (Biofa AG, Germany) over a longer time period (21 d), but similarly under just one set of environmental conditions. Since environmental conditions like temperature and air humidity are variable in time and space, evaluating changes in substance effectiveness for several of their combinations would offer more practical insights.

The aim of this study was to evaluate the insecticidal efficacy of wood ash from different sources and the diatomaceous earth product SilicoSec against *S. granarius* (both adults and progeny) under varying environmental conditions. Specifically, the study sought to determine the lethal concentration metrics (LC_50_ and LC_90_) using Probit analysis, assess the influence of temperature, relative humidity (RH), and exposure duration on treatment effectiveness, and determine a reliable range of wood ash concentrations to provide a cost-effective, locally available alternative to diatomaceous earth products like SilicoSec for pest control in grain storage facilities.

## Materials and Methods

### Insects

Granary weevil (*S. granarius*) adults were reared in plastic boxes (length:width:height = 30:24:12 cm) in growing chambers (producer: Kambič Laboratory equipment, Semič, Slovenia) in the Laboratory of Entomology (Chair for Phytomedicine, Agricultural Engineering, Crop Production, Pasture and Grassland Management, Biotechnical Faculty, Ljubljana) at temperature 22 ± 2 °C, RH 55 ± 5%, and continuous darkness, using wheat grains as their food. Ten years prior study, *S. granarius* adults were obtained from Pesticide and Environment Research Institute from Belgrade (Serbia).

### Wood Ash Preparation

Our study was based on testing the efficacy of different inert dusts against granary weevil adults. We have tested the efficacy of 3 different wood ashes and a commercial diatomaceous earth product. Five hundred grams lots of wheat grain were used for specific treatment. Wood ashes were applied in 7 different concentrations, respectively 0.0385, 0.077, 0.15, 0.31, 0.625, 1.25, and 2.5 w% (as weight %). We used wood ash from Norway spruce (*Picea abies* [L.] Karsten) and European silver fir (*Abies alba* [Mill.]). The wood used for preparing the ash was acquired in 3 locations—for spruce in Zgornja Lipnica (46.323130, 14.186794) and in Gorenje pri Zrečah (46.400031, 15.385970); for fir in Zgornja Lipnica. The wood was air-dried for several months in a shadowed place before being burned. Wood ashes were sieved through 1 mm mesh and stored in 1 liter plastic jars with wide neck, then kept in room conditions in darkness, until needed for bioassay. Diatomaceous earth product SilicoSec (Biofa AG, Germany) served as the positive control and untreated grain as negative control.

### Granulometry and Geochemical Analysis of Wood Ashes

For granulometric analysis, the Fritsch Analysette 22-28 device was used, which combines a laser granulometer and dynamic image analysis. The size range of measurements with the mentioned equipment varies between 0.01 and 2,100 µm. Due to the larger amount of the sample, the latter was quartered until a representative sample was obtained for further analysis (approx. 1 to 2 g). The sample was then mixed with distilled water and put into ultrasonic bath for 10 min to break up any agglomerates and create an even suspension. The suspension was then measured with a laser granulometer. Each individual sample measurement was performed 3 times to ensure the representativeness of the measured data. From all measured curves of individual samples, a representative average curve was created, in which all measured fractions and their relative representation were considered.

Inductively Coupled Plasma Emission Spectroscopy (ICP-ES) and Inductively Coupled Plasma Mass Spectroscopy (ICP-MS) were used to perform the geochemical analysis of the dusts.

### Bioassay

Bioassays were conducted to evaluate the efficacy of different treatments (JeL—white fir ash from Jelovica, SiS—SilicoSec, SmL—Norway spruce ash from Zgornja Lipnica, and SmZ—Norway spruce ash from Gorenje nad Zrečami) on mortality rates under controlled conditions. Wheat grain for specific treatment (500 g) was placed into 1,000 ml Erlenmayer flasks, and different powders concentrations were added to each. GFL overhead rotator 3040 (supplier: ProfiLab 24, Berlin, Germany) was used for mixing wheat grain with powders. Growing chambers (producer: Kambič Laboratory Equipment, Semič, Slovenija) were kept at 4 temperature levels (15, 20, 25, 30 °C), and 2 RH levels (55%, 75%). The beetles were counted on multiple timepoints (7, 14, and 21 d), and the data were collected as dose–response values. Establishment of individual treatments (mixing powders with grain) was taken from previously described methodology by Bohinc et al. (2018) and Bohinc et al. (2020). Each specific treatment (500 g of wheat grain) was divided into 9 Erlenmayer flasks (100 ml); all bioassays were repeated 3 times (3 series of Erlenmayer flasks) by preparing new treated Erlenmayer flasks each time (3 × 3 Erlenmayer flasks for each combination). Thirty unsexed individuals were introduced into one 100 ml Erlenmayer flask, respectively. Erlenmayer flasks were covered with mesh, to prevent insects from escaping.

In total, our research was based on 9 replications of 7 doses/concentrations of 4 inert dusts under 2 RH and 4 temperature conditions. The experimental factors were concentration, treatment (type of inert product), RH, and temperature. Evaluation variables were granary weevil counts on 3 separate dates (exposure intervals). Probit analysis was performed to estimate lethal concentration metrics (LC_50_ and LC_90_).

After the 21-d adult mortality experiment, wheat from each treatment (potentially containing eggs) was transferred to separate containers. These containers were all maintained under uniform room conditions (22 ± 2 °C, 60 ± 5% RH) for 60 d. After this period, emerged beetles (both dead and alive) were counted.

### Data Analysis

Mortality data were adjusted for control mortality using Abbott’s (1925) formula when control mortality exceeded 5%. Results were expressed as percentages. Probit analysis was conducted in RStudio (Posit Team, 2024) using the “MASS” and “drc” packages for probit regression, standard error (SE) analysis, and lethal concentration (LC) calculations. Estimates for LC_50_ and LC_90_ concentrations were obtained from probit modeling. The dataset was refined to remove entries where all datapoints showed 100% mortality, values deemed unrealistically high (LC estimates > 2.5 g/100 g), infinite values arising from model limitations in handling extreme values. Specific treatment combinations (substance, timepoint, temperature, and RH) excluded due to 100% mortality in Abbott’s corrected data are listed in Appendix Table 3. For these combinations, even the lowest dusts concentrations were too high to allow for a LC estimation with the Probit model.

ANOVA was conducted separately for LC₅₀ and LC₉₀ to assess the effects of treatment, timepoint, temperature, and RH on mortality. Additionally, confidence intervals (CI) were used for another level of insight into possible differences. Tukey’s Honest Significant Difference (HSD) test was used for posthoc comparisons. ANOVA assumptions were verified using residual diagnostic plots. ANOVA on Total Emerged Beetles was executed to evaluate treatment effects on egg laying in the initial adult mortality phase. ANOVA on proportion of dead weevils—analyzed treatment effectiveness in killing emerged weevils. Since the results were highly skewed (proportions close to 0 or 1), beta regression was applied, using the “betareg” package in R to model the proportion of dead beetles as a continuous variable constrained between 0 and 1, considering both mean and dispersion.

## Results

### Granulometry and Geochemical Analysis

Morphological results revealed some heterogeneity between the size of SiS and wood ash particles. For SiS, the particle size according to granulometric analysis was 14.70 ± 1.85 µm, while for JeL, SmL, and SmZ, they were measured at 500.10 ± 183.58, 196.43 ± 99.90, 160.51 ± 58.25 µm, respectively. Results of the geochemical analysis are presented in Table 1. They confirm that SiS is mostly made of SiO_2_ (79.5%), while SiO_2_ in wood ashes ranges only from 12.8% to 17.5%. The largest component of the wood ashes was CaO, amounting to 31.0% to 37.6% of the total. For SiS, CaO content was less than 0.5%.

**Table 1.** Geochemical analysis results for the 4 treatments

Analyte	Relative content (%)			
	**SiS**	**JeL**	**SmL**	**SmZ**
**SiO**_**2**_	**79.48**	17.49	13.46	12.83
Al_2_O_3_	4.62	5.11	4.25	4.23
Fe_2_O_3_(T)	2	2.55	1.34	2.19
MnO	0.029	0.987	1.801	1.765
MgO	0.33	3.91	4.3	5.78
**CaO**	0.45	**31.04**	**37.05**	**37.55**
Na_2_O	0.11	0.73	0.53	0.59
K_2_O	0.15	9.33	7.42	8.61
TiO_2_	0.154	0.453	0.218	0.304
P_2_O_5_	0.02	2.33	2.74	2.23
Loss on ignition	11.18	25.37	26.34	21.43
**Total**	98.52	99.29	99.46	97.51

### Adults

**Fig. 1.**
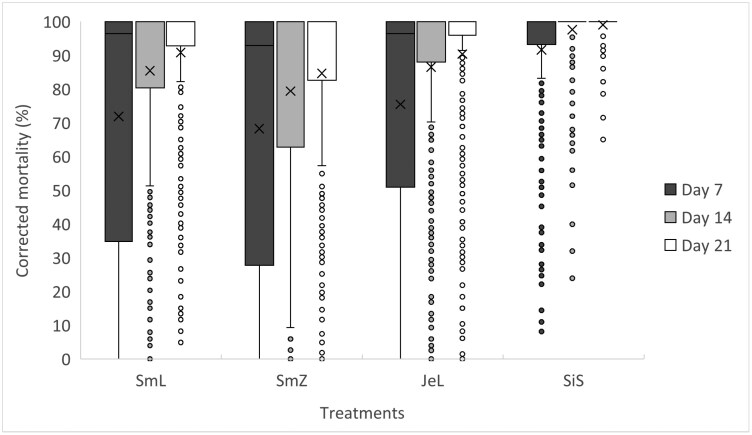
Distribution plot of *S. granarius* adults corrected mortality results for the different treatments and exposure intervals (counting days), but with generalized data for temperature and RH (the “X” represents the median and “–“ the average values, SmL stands for Norway spruce wood ash from Zgornja Lipnica, SmZ stands for Norway spruce wood ash from Gorenje nad Zrečami, JeL stands for white fir wood ash, SiS stands for SilicoSec).

A general overview of the treatments’ influence on the Abbott’s corrected mortality (not differentiating between temperature and RH combinations) over several weeks can be seen in Fig. 1. There is a visible difference between the positive control—SilicoSec (SiS) treatment and the 3 wood ash treatments. While the average value for corrected mortality of all treatments exceeded 90% in all cases, it needs to be noted that the quartile ranges for the wood ash treatments are much larger, indicating a much greater variability between the individual treatment concentrations and temperature–RH conditions.

**Fig. 2.**
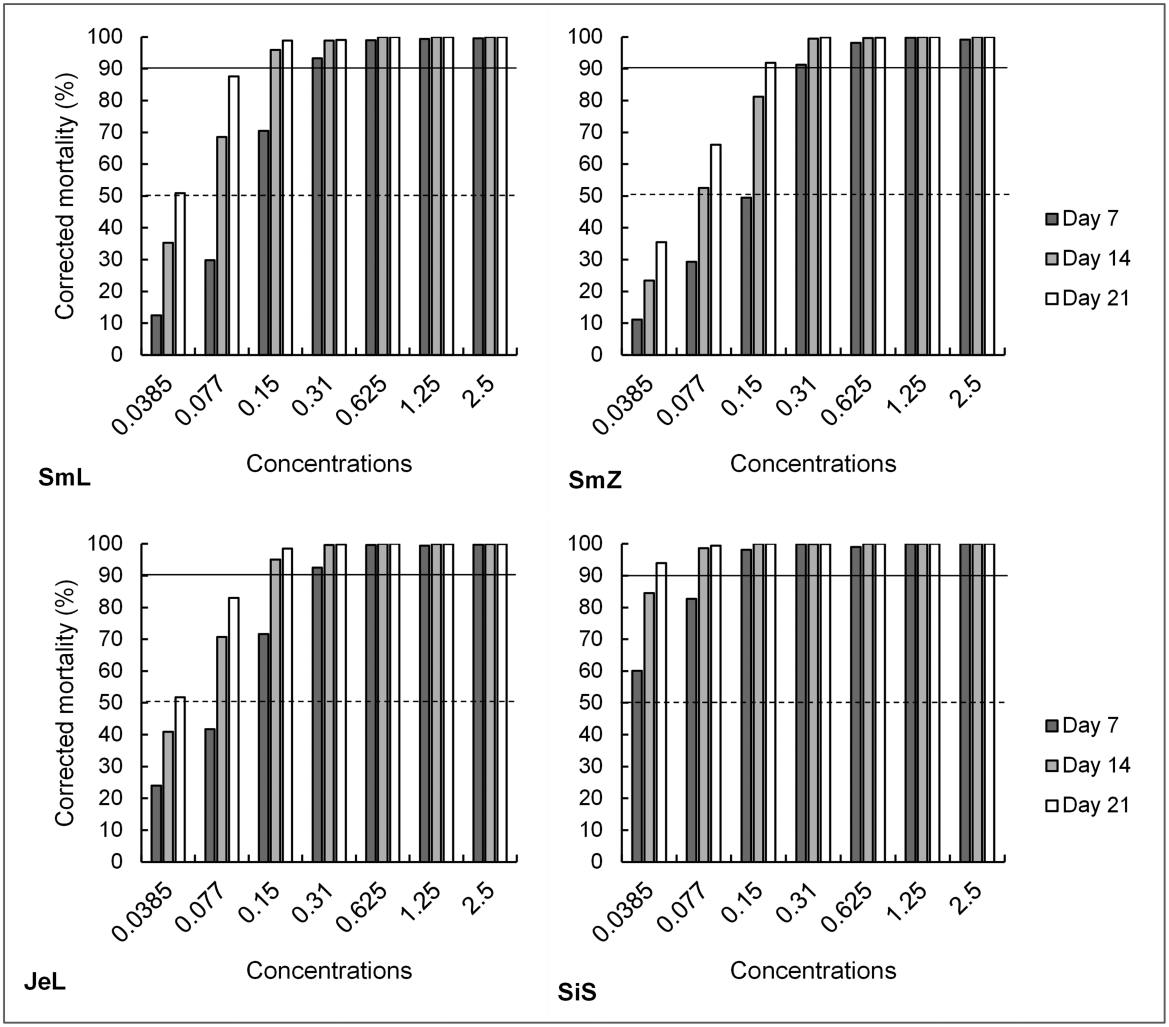
Corrected mortality of the *S. granarius* adults based on the different concentrations (w%) for the 4 treatments, but with generalized data for temperature and RH (SmL stands for Norway spruce wood ash from Zgornja Lipnica, SmZ stands for Norway spruce wood ash from Gorenje nad Zrečami, JeL stands for white fir wood ash, SiS stands for SilicoSec) at different exposure intervals.

With wood ashes, the correct concentration was of crucial importance to achieve sufficient mortality, which can also be seen in Fig. 2. There, visual estimation could already give us a basic feel for the needed concentrations.

For greater clarity, the lethal concentrations (LC_50_ and LC_90_) for each treatment–environmental factor combination were estimated by Probit modeling. LC_50_ values are more relevant for comparison, but the LC_90_ values are presented as well to provide a broader scope. The general analysis of the Probit-estimated concentrations confirmed that mortality increases at higher concentrations. According to the results, the increase factor is 2.77, meaning that with each doubling of concentration, mortality increases by a factor of 2.77 (*Z* = 63.03; df = 503; *P* < 2^−^16).

The general ANOVA between treatment, temperature, and RH classes has revealed that the differences between RH classes were not statistically significant neither for LC_50_ (*F* = 2.68, df = 1, *P* = 0.1) nor LC_90_ (*F* = 0.11, df = 1, *P* = 0.7), while the differences for temperature classes were only statistically significant (*F* = 3.31, df = 3, *P* = 0.026*) in the case of LC_90_ and not LC_50_ (*F* = 2.64, df = 3, *P* = 0.057). Upon further investigation using the Tukey HSD test, no strong evidence was found to suggest statistical differences between the temperature classes (*P* > 0.05), so the factors of RH and temperature were both excluded from further analysis. The full results of the Probit-estimated concentrations for LC_50_ and LC_90_ are listed in the appendices (Appendix Table 4). Corrected mortality diagrams, showing the influence of treatments and their individual concentrations, can be seen in Fig. 2. The differences between treatment classes were statistically significant for both LC_50_ (*F* = 5.22, df = 3, *P* = 0.00269**) and LC_90_ (F = 3.66, df = 3, *P* = 0.0167*). Treatments comparison based on the posthoc test results for LC_50_ and LC_90_ are presented in Fig. 4. The Tukey HSD test highlights significant differences in only 2 treatment combinations. SmZ-SiS has a statistically significant difference in means in both LC_50_ (difference = 0.055 ± 0.041, *P* = 0.004) and LC_90_ (difference = 0.214 ± 0.187, *P* = 0.018) cases.

**Fig. 3.**
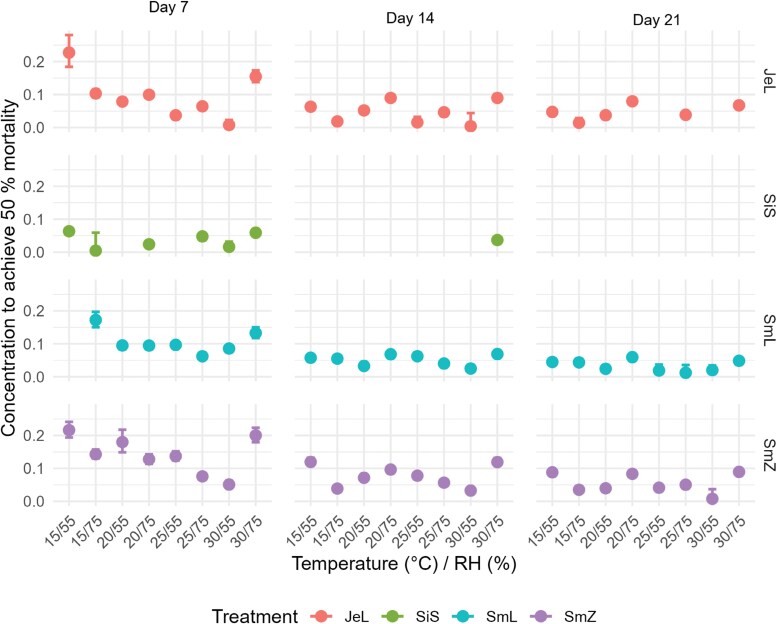
LC_50_ values for all combinations of treatments with the corresponding CIs.

In order to gain further insight into possible differences, CI for the LC_50_ were compared for the different treatments. Results are presented in Fig. 3, while the treatment combinations, for which significant differences were determined by the CIs are listed in the appendices (Appendix Table 5).

### Progeny Experiment

Average numbers of live and dead beetles for each treatment from the progeny experiment are presented in Fig. 5. ANOVA for live and dead beetle counts for the different temperature and RH classes revealed (Table 2) all variables (Treatment: *F* = 28.5, df = 3, *P* < 0.00001; Temperature: *F* = 24.3, df = 2, *P* < 0.00001; RH: *F* = 15.2, df = 1, *P* < 0.00001) to have a statistically significant difference, while of the second-order interactions only Treatment:Temperature:RH combination was significant (*F* = 8.7, df = 3, *P* = 0.0001).

**Table 2.** ANOVA results for live and dead beetles counts for different temperature and RH classes. The posthoc test used for means separation was the Tukey HSD test

Response: Emergence	*F* value	df	*P*
Treatment	131.155	4	<2.2e−16 ***
Concentration	38.9235	1	7.980e−10 ***
Temp	115.8128	1	<2.2e−16 ***
RH	20.0464	1	8.938e−06 ***
Treatment:Concentration	2.9447	3	0.0323479 *
Treatment:Temp	102.0604	4	<2.2e−16 ***
Concentration:Temp	19.1271	1	1.426e−05 ***
Treatment:RH	6.8826	4	1.987e−05 ***
Concentration:RH	6.7889	1	0.0093844 **
Temp:RH	10.525	1	0.0012385 **
Treatment:Concentration:Temp	1.0698	3	0.3612566
Treatment:Concentration:RH	0.4179	3	0.7402107
Treatment:Temp:RH	5.7794	4	0.0001422 ***
Concentration:Temp:RH	0.9539	1	0.3290942
Treatment:Concentration:Temp:RH	0.2793	3	0.8403509

## Discussion

### Adults

An interesting discovery is that the wood ash was more effective than in a previous study (Bohinc and Trdan 2017), where common beech (*Fagus sylvatica* L.) ash at 2.5 g/100 g after 7 d, resulted in a mortality of merely 50%, not well over 90%, like in this case, emphasizing the idea that different sources of ash could yield different results.

General analysis of the Probit-estimated concentrations confirmed that mortality increases at higher concentrations (LC_50_: *F* = 5.22, df = 3, *P* = 0.00269**; LC_90_: *F* = 3.66, df = 3, *P* = 0.0167*), which is logical and was reported previously (Shams et al. 2011). Nonetheless, ANOVA results for the different factors slightly contradict some previous studies (Vavias and Athanassiou 2004), where temperature and RH factors were found to be influential on the effectiveness of insecticidal dusts. In our study, these effects could not be confirmed. What could be the reason for this discrepancy is that the concentrations of the dusts used were rather high (visible in Fig. 2), which has possibly diminished the environmental effects in this particular study.

**Fig. 4.**
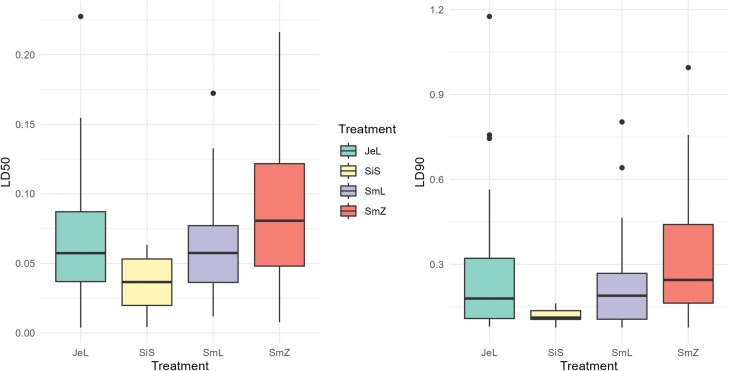
LC_50_ and LC_90_ results for *S. granarius* adults for the 4 treatments (SmL stands for Norway spruce wood ash from Zgornja Lipnica, SmZ stands for Norway spruce wood ash from Gorenje nad Zrečami, JeL stands for white fir wood ash, SiS stands for SilicoSec) as estimated by the Probit model (vertical axis represents the estimated concentrations). Data for temperature and RH were generalized in the figure, but the full calculated data are shown in Appendix Table 4.

Results of the Tukey HSD test for the 4 treatments (Fig. 4) suggest that the source location of wood used to create ash might be more influential on the insecticidal effects than wood species LC_50_: *F* = 5.22, df = 3, *P* = 0.00269**; LC_90_; *F* = 3.66, df = 3, *P* = 0.0167*). Spruce and fir ash from Lipnica had quite similar average values and intervals, while the spruce ash from Zreče showed a high variability and a much lower effectiveness. Our initial thought was that this effect could be connected to the geochemical composition of the different samples, based on the soil mineral composition in different locations. The wood ash geochemical analysis results, on the other hand, suggest greater similarities between the 2 spruce samples than between the ashes from the same location. The only analyte where samples were more similar based on location was the loss of ignition (LOI), which refers to the amount of weight loss a sample undergoes when it is heated to a high temperature. Despite the possibly important implications of this phenomenon, scarce past studies (Rojht et al. 2010) show a lack of in-depth explanation, so additional experiments would be needed in this field.

Probit-estimated LC_90_ values (Fig. 4) were consistently higher than LC_50_, which is logical. It is interesting that the size of the intervals for LC_50_ and LC_90_ differed greatly for wood ash treatments, indicating their variable effectiveness. SilicoSec results, on the other hand, were very consistent. In the official documentation for SilicoSec (Fito-info 2025), a dosage of 0.1 to 0.2 g/100 g is recommended for treatment of grain in storage facilities. Similar concentrations are proposed by other authors (Traian et al. 2015, Mortazavi et al. 2024). When comparing this to the Probit-estimated values from our experiment, we can find out that the officially recommended values (Fito-info 2025) are at least 2 times higher than what was found to be sufficient in our case. After 7 d, the Probit-estimated LC_90_ concentration was determined at 0.098 ± 0.005 g/100 g, which is roughly the low recommended amount, but after 21 d, the LC_90_ Probit-estimated value dropped down to just 0.032 ± 0.003, which is a third of the lower recommended value. This finding suggests that even lower dosages of SilicoSec could be effective at suppressing *S. granarius* infestations in storage facilities. The narrow quartile range, indicating a consistency in effectiveness, further emphasizes this claim, especially when aiming at longer-term storage.

### Progeny Experiment

**Fig. 5.**
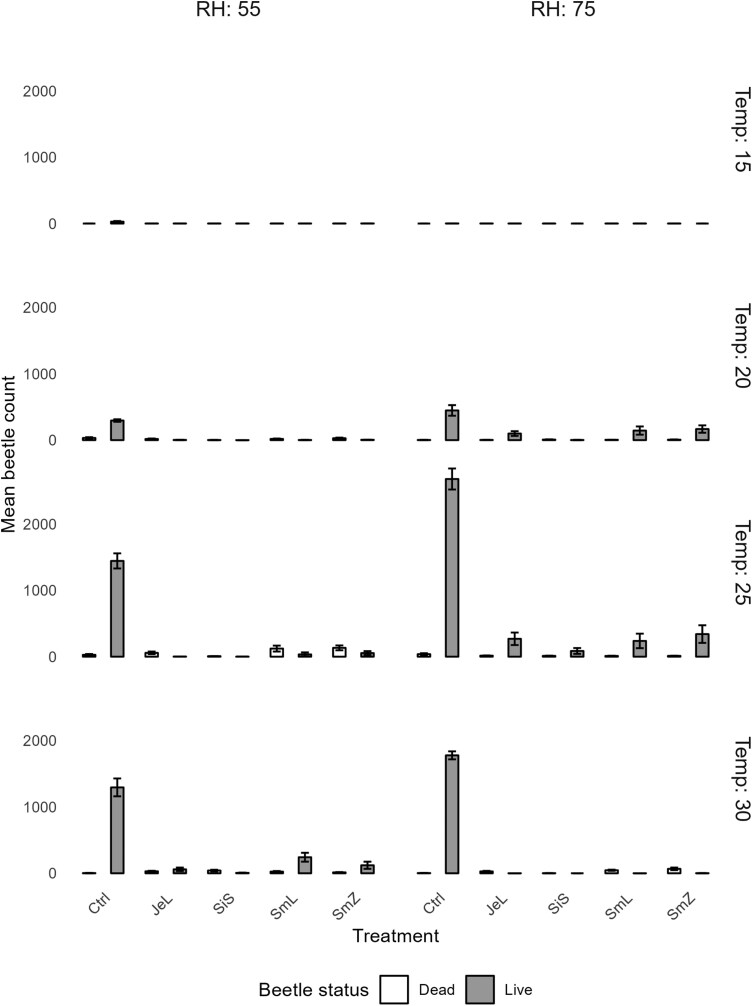
Results of the progeny experiment: average number of live and dead *S. granarius* adults counted after 60 d for the generalized concentrations for each of the treatments for the different temperature and RH classes.

The finding that all factors were found to have statistically significant influence on the progeny experiment results (Treatment: *F* = 28.5, df = 3, *P* < 0.00001; Temperature: *F* = 24.3, df = 2, *P* < 0.00001; RH: F = 15.2, df = 1, *P* < 0.00001) is somewhat surprising, since the temperature and RH factors were not found to be significant in the first part of the experiment on adults. Even though environmental conditions showed little influence on mortality of adults, they might be quite influential on reproduction and egg laying. This effect can be observed in Fig. 5. At low temperatures the beetles, even in the nontreated control (Ctrl) did not lay many eggs (highest count was 61 adult weevils). The weevils seem to have been most active at 25 °C, and more so at the higher RH, which is in accordance with past studies (Omar et al. 2014). The highest number of live weevils (3,197) was recorded in the Ctrl treatment, 25 °C and 75% RH combination.

The highest numbers for other treatments in the same conditions were nearly half as low, while the average was almost 10 times lower, indicating that the treatments were considerably more effective. At 30 °C the numbers dropped again, but they were still higher than at 20 °C. Quite interestingly, the 25 °C–55% RH and 30 °C–75% RH were the only combinations where the number of dead beetles was not only noticeable but was higher than that of the live ones (except for the Ctrl treatment). These findings strengthen the thesis (Omar et al. 2014) that storage conditions have a significant influence on the effects of different treatments against the *S. granarius* adults egg laying activity, but also by interferences on the morphological structures of the beetles’ genital apparatuses, which in turn affect mating and the number of eggs laid.

To investigate further, a comparison of the proportion between live and dead beetles based on the Beta regression model was performed to better understand why in some cases we found more dead and in other more live beetles. Significant effects (Beta regression’s Logit model) results show that treatments JeL (*P* = 9.09e−05), SiS (*P* = 0.000227), SmL (*P* = 0.001231), and SmZ (*P* = 0.004989) all significantly affect the proportion of dead beetles. Temperature (*P* < 2e*−*16) is also highly significant in explaining the proportion of dead beetles, while RH and concentration did not show significant effects (*P* > 0.05 for both). RH was already found to be less significant than temperature in past studies (Khan 2023). Susceptibility of young eggs, prepupae, and pupae stages to lower temperatures (15 °C) was reported in previous studies (Howe and Hole 1968, Omar et al. 2014), explaining why progeny emergence was very low at lower temperatures. We were unable to find additional explanations in past studies; thus, we conclude that selection of the right treatment in combination with the correct temperature range in storage facilities is crucial for effective control of *S. granarius* offspring.

This research uniquely evaluates the efficacy of wood ash as a locally sourced, cost-effective alternative to commercial diatomaceous earth (SilicoSec) for controlling *S. granarius* in grain storage facilities. By focusing on different wood ash treatments and their optimal dosages, the study provides practical insights for sustainable pest management.

The primary hypothesis posited that wood ash could serve as an effective alternative to SilicoSec in managing *S. granarius* populations. This hypothesis was supported, as wood ash treatments achieved mortality rates exceeding 90% under certain conditions. Additionally, it was hypothesized that environmental factors such as temperature and RH would significantly influence treatment efficacy. However, the study found conflicting evidence regarding temperature and RH significance. While they did not show a statistically significant impact on adults’ mortality, they were significantly influential for progeny emergence, leading to the partial rejection of the secondary hypothesis.

Despite the promising results, there is still uncertainty regarding the variability in the effectiveness of different wood ash samples. Findings suggest that factors such as particle size and chemical composition may influence outcomes, but they are rarely researched in this context. The exclusion of temperature and RH as significant factors may also be context-dependent, warranting caution in generalizing these findings across different storage environments. This is, however, a very important topic to investigate in future studies to arrive at rational considerations.

This research contributes to the body of knowledge on alternative pest control methods by providing empirical evidence supporting the use of wood ash in grain storage. It offers comparative analysis with a commercial product, highlighting the potential for integrating locally available materials into pest management strategies (Trematerra and Fleurat-Lessard 2015). For effective implementation, it is recommended to determine the appropriate dosage of wood ash for specific storage conditions, considering factors such as grain type and storage duration. The study suggests that, in some cases, the recommended dosages for SilicoSec could be reduced by a factor of 2 to 3 when used against *S. granarius*, especially for longer-term storage, making pest control more economical. Policymakers are encouraged to promote the use of locally sourced materials like wood ash in integrated pest management programs. This approach can reduce reliance on commercial pesticides, lower costs for farmers, and support sustainable agricultural practices.

Further research is needed to explore the underlying factors contributing to the variability in wood ash efficacy, such as the influence of different tree species and combustion processes. Additionally, large scale trials under diverse environmental conditions would help validate the laboratory findings and refine application guidelines for broader adoption.

## Supplementary material

Supplementary material is available at *Journal of Economic Entomology* online.

*Conflicts of interest.* None declared.

toaf109_suppl_Supplementary_Appendix

## Data Availability

The datasets generated during and/or analyzed during the current study are available from the corresponding author on reasonable request.
