# Supplementary material for: Efficacy of wood ash and diatomaceous earth against Sitophilus granarius: influence of dose, environmental conditions, and geomorphological composition
Source: J Econ Entomol. 2025 Jun 12;118(4):1972–80. doi: 10.1093/jee/toaf109 (PMC12397965; doi:10.1093/jee/toaf109)
Supplement: toaf109_suppl_Supplementary_Appendix [file toaf109_suppl_supplementary_appendix.docx]

**Appendices**

Table 3: Treatments excluded from ANOVA because of extremely high estimated mortality rates

| **Treatment** | **Timepoint** | **Temperature** | **Relative humidity** |
| --- | --- | --- | --- |
| SmL | 7 | 15 | 55 |
| JeL | 21 | 25 | 55 |
| JeL | 21 | 30 | 55 |
| SiS | 7 | 20 | 55 |
| SiS | 7 | 25 | 55 |
| SiS | 14 | 15 | 55 |
| SiS | 14 | 20 | 75 |
| SiS | 14 | 20 | 55 |
| SiS | 14 | 25 | 75 |
| SiS | 21 | 15 | 55 |
| SiS | 21 | 20 | 75 |
| SiS | 21 | 25 | 75 |
| SiS | 21 | 30 | 75 |

SmL – Norway spruce ash from Lipnica; SmZ - Norway spruce ash from Zreče; JeL – white fir ash from Zgornja Lipnica; SiS – SilicoSec

Table 4: Probit estimated concentrations (g/100g) for LC_50_ and LC_90_

| **Treatment** | **Timepoint** | | **Temperature** | | **RH** | | **n** | | **Slope SE** | | **LC50** | **LC50 CI** | **LC90** | **LC90 CI** | **Chi_Square** |
| --- | --- | --- | --- | --- | --- | --- | --- | --- | --- | --- | --- | --- | --- | --- | --- |
| SmL | 7 | | 15 | | 75 | | 23 | | 0.975 ± 0.097 | | 0.172 | (0.151, 0.197) | 0.641 | (0.449, 0.916) | 44.855 |
| SmL | 7 | | 15 | | 55 | | 25 | | 0.397 ± 0.096 | | 0.514 | (0.212, 1.246) | 13.003 | (1.189, 142.192) | 39.98 |
| SmL | 7 | | 20 | | 75 | | 24 | | 1.049 ± 0.095 | | 0.095 | (0.086, 0.104) | 0.321 | (0.25, 0.412) | 401.638 |
| SmL | 7 | | 20 | | 55 | | 25 | | 1.179 ± 0.097 | | 0.095 | (0.087, 0.103) | 0.282 | (0.229, 0.347) | 68.253 |
| SmL | 7 | | 25 | | 75 | | 18 | | 2.017 ± 0.174 | | 0.062 | (0.059, 0.066) | 0.117 | (0.103, 0.134) | 35.64 |
| SmL | 7 | | 25 | | 55 | | 39 | | 0.979 ± 0.056 | | 0.097 | (0.089, 0.105) | 0.358 | (0.306, 0.419) | 538.397 |
| SmL | 7 | | 30 | | 75 | | 36 | | 0.712 ± 0.052 | | 0.133 | (0.117, 0.15) | 0.803 | (0.632, 1.021) | 66.507 |
| SmL | 7 | | 30 | | 55 | | 31 | | 1.172 ± 0.07 | | 0.086 | (0.079, 0.093) | 0.255 | (0.222, 0.294) | 67.144 |
| SmL | 14 | | 15 | | 75 | | 18 | | 1.749 ± 0.166 | | 0.055 | (0.052, 0.059) | 0.114 | (0.098, 0.133) | 26.562 |
| SmL | 14 | | 15 | | 55 | | 27 | | 1.067 ± 0.089 | | 0.058 | (0.052, 0.063) | 0.191 | (0.159, 0.23) | 69.009 |
| SmL | 14 | | 20 | | 75 | | 21 | | 2 ± 0.143 | | 0.068 | (0.064, 0.072) | 0.129 | (0.115, 0.145) | 32.495 |
| SmL | 14 | | 20 | | 55 | | 20 | | 0.668 ± 0.125 | | 0.033 | (0.025, 0.043) | 0.224 | (0.133, 0.377) | 88.916 |
| SmL | 14 | | 25 | | 75 | | 14 | | 1.515 ± 0.209 | | 0.04 | (0.036, 0.044) | 0.093 | (0.076, 0.114) | 14.168 |
| SmL | 14 | | 25 | | 55 | | 19 | | 0.638 ± 0.133 | | 0.062 | (0.053, 0.074) | 0.465 | (0.195, 1.106) | 33.223 |
| SmL | 14 | | 30 | | 75 | | 28 | | 1.099 ± 0.077 | | 0.069 | (0.063, 0.076) | 0.221 | (0.189, 0.258) | 113.29 |
| SmL | 14 | | 30 | | 55 | | 18 | | 0.781 ± 0.17 | | 0.025 | (0.018, 0.035) | 0.128 | (0.085, 0.194) | 61.329 |
| SmL | 21 | | 15 | | 75 | | 17 | | 2.025 ± 0.186 | | 0.044 | (0.041, 0.047) | 0.082 | (0.074, 0.092) | 11.614 |
| SmL | 21 | | 15 | | 55 | | 21 | | 1.247 ± 0.127 | | 0.045 | (0.041, 0.049) | 0.125 | (0.105, 0.15) | 39.246 |
| SmL | 21 | | 20 | | 75 | | 17 | | 2.523 ± 0.188 | | 0.059 | (0.056, 0.062) | 0.099 | (0.09, 0.108) | 17.709 |
| SmL | 21 | | 20 | | 55 | | 16 | | 0.919 ± 0.192 | | 0.024 | (0.017, 0.033) | 0.097 | (0.071, 0.133) | 66.3 |
| SmL | 21 | | 25 | | 75 | | 12 | | 0.691 ± 0.288 | | 0.012 | (0.004, 0.035) | 0.077 | (0.046, 0.128) | 16.012 |
| SmL | 21 | | 25 | | 55 | | 15 | | 0.553 ± 0.189 | | 0.019 | (0.009, 0.037) | 0.19 | (0.075, 0.479) | 15.932 |
| SmL | 21 | | 30 | | 75 | | 25 | | 0.937 ± 0.082 | | 0.048 | (0.043, 0.055) | 0.19 | (0.154, 0.234) | 149.695 |
| SmL | 21 | | 30 | | 55 | | 11 | | 0.95 ± 0.34 | | 0.02 | (0.012, 0.034) | 0.077 | (0.049, 0.121) | 27.614 |
| **Treatment** | **Timepoint** | | **Temperature** | | **RH** | | **n** | | **Slope SE** | | **LC50** | **LC50 CI** | **LC90** | **LC90 CI** | **Chi_Square** |
| SmZ | 7 | 15 | | 75 | | 36 | | 0.866 ± 0.055 | | 0.143 | | (0.13, 0.157) | 0.628 | (0.503, 0.785) | 148.878 |
| SmZ | 7 | 15 | | 55 | | 19 | | 1.334 ± 0.12 | | 0.216 | | (0.194, 0.241) | 0.565 | (0.442, 0.723) | 107.533 |
| SmZ | 7 | 20 | | 75 | | 27 | | 1.047 ± 0.093 | | 0.128 | | (0.114, 0.143) | 0.435 | (0.323, 0.584) | 38.245 |
| SmZ | 7 | 20 | | 55 | | 23 | | 0.892 ± 0.108 | | 0.18 | | (0.149, 0.218) | 0.757 | (0.458, 1.253) | 56.096 |
| SmZ | 7 | 25 | | 75 | | 23 | | 1.615 ± 0.113 | | 0.076 | | (0.071, 0.081) | 0.168 | (0.149, 0.189) | 38.494 |
| SmZ | 7 | 25 | | 55 | | 38 | | 0.798 ± 0.051 | | 0.137 | | (0.124, 0.152) | 0.684 | (0.539, 0.869) | 138.879 |
| SmZ | 7 | 30 | | 75 | | 37 | | 0.8 ± 0.049 | | 0.2 | | (0.18, 0.223) | 0.995 | (0.816, 1.213) | 110.912 |
| SmZ | 7 | 30 | | 55 | | 27 | | 0.582 ± 0.061 | | 0.051 | | (0.041, 0.063) | 0.46 | (0.323, 0.655) | 103.915 |
| SmZ | 14 | 15 | | 75 | | 23 | | 0.789 ± 0.098 | | 0.039 | | (0.032, 0.046) | 0.196 | (0.145, 0.265) | 49.645 |
| SmZ | 14 | 15 | | 55 | | 21 | | 1.11 ± 0.122 | | 0.12 | | (0.108, 0.133) | 0.38 | (0.28, 0.515) | 27.935 |
| SmZ | 14 | 20 | | 75 | | 27 | | 1.215 ± 0.092 | | 0.096 | | (0.089, 0.105) | 0.277 | (0.228, 0.337) | 72.822 |
| SmZ | 14 | 20 | | 55 | | 24 | | 1.123 ± 0.098 | | 0.072 | | (0.066, 0.078) | 0.224 | (0.181, 0.279) | 46.855 |
| SmZ | 14 | 25 | | 75 | | 15 | | 2.458 ± 0.196 | | 0.057 | | (0.054, 0.06) | 0.095 | (0.087, 0.105) | 20.145 |
| SmZ | 14 | 25 | | 55 | | 26 | | 0.978 ± 0.089 | | 0.078 | | (0.071, 0.086) | 0.289 | (0.223, 0.373) | 40.321 |
| SmZ | 14 | 30 | | 75 | | 20 | | 1.13 ± 0.107 | | 0.119 | | (0.108, 0.131) | 0.371 | (0.298, 0.461) | 51.673 |
| SmZ | 14 | 30 | | 55 | | 16 | | 1.047 ± 0.182 | | 0.033 | | (0.027, 0.039) | 0.112 | (0.083, 0.15) | 49.94 |
| SmZ | 21 | 15 | | 75 | | 20 | | 0.958 ± 0.119 | | 0.035 | | (0.03, 0.042) | 0.134 | (0.106, 0.17) | 33.873 |
| SmZ | 21 | 15 | | 55 | | 23 | | 1.388 ± 0.11 | | 0.088 | | (0.082, 0.095) | 0.222 | (0.189, 0.26) | 23.803 |
| SmZ | 21 | 20 | | 75 | | 24 | | 1.33 ± 0.102 | | 0.084 | | (0.077, 0.09) | 0.219 | (0.184, 0.261) | 51.8 |
| SmZ | 21 | 20 | | 55 | | 21 | | 0.961 ± 0.114 | | 0.04 | | (0.034, 0.046) | 0.15 | (0.118, 0.192) | 41.11 |
| SmZ | 21 | 25 | | 75 | | 17 | | 2.443 ± 0.186 | | 0.05 | | (0.048, 0.053) | 0.085 | (0.078, 0.093) | 31.839 |
| SmZ | 21 | 25 | | 55 | | 17 | | 0.786 ± 0.164 | | 0.041 | | (0.035, 0.049) | 0.211 | (0.117, 0.38) | 30.708 |
| SmZ | 21 | 30 | | 75 | | 21 | | 1.176 ± 0.123 | | 0.089 | | (0.081, 0.098) | 0.266 | (0.216, 0.328) | 154.606 |
| SmZ | 21 | 30 | | 55 | | 13 | | 0.559 ± 0.234 | | 0.008 | | (0.002, 0.037) | 0.077 | (0.049, 0.12) | 5.668 |
| JeL | 7 | 15 | | 75 | | 32 | | 0.754 ± 0.062 | | 0.103 | | (0.092, 0.115) | 0.564 | (0.415, 0.767) | 46.046 |
| **Treatment** | **Timepoint** | **Temperature** | | **RH** | | **n** | | **Slope SE** | | **LC50** | | **LC50 CI** | **LC90** | **LC90 CI** | **Chi_Square** |
| JeL | 7 | 15 | | 55 | | 25 | | 0.78 ± 0.09 | | 0.227 | | (0.184, 0.281) | 1.176 | (0.676, 2.046) | 72.357 |
| JeL | 7 | 20 | | 75 | | 15 | | 1.895 ± 0.163 | | 0.099 | | (0.092, 0.107) | 0.196 | (0.174, 0.22) | 31.577 |
| JeL | 7 | 20 | | 55 | | 24 | | 1.685 ± 0.112 | | 0.079 | | (0.074, 0.084) | 0.169 | (0.15, 0.189) | 19.65 |
| JeL | 7 | 25 | | 75 | | 23 | | 1.047 ± 0.102 | | 0.064 | | (0.058, 0.071) | 0.218 | (0.171, 0.279) | 51.15 |
| JeL | 7 | 25 | | 55 | | 31 | | 0.558 ± 0.059 | | 0.037 | | (0.029, 0.047) | 0.366 | (0.261, 0.515) | 67.98 |
| JeL | 7 | 30 | | 75 | | 28 | | 0.807 ± 0.07 | | 0.155 | | (0.138, 0.173) | 0.757 | (0.571, 1.004) | 92.604 |
| JeL | 7 | 30 | | 55 | | 27 | | 0.279 ± 0.062 | | 0.008 | | (0.003, 0.023) | 0.745 | (0.271, 2.046) | 82.207 |
| JeL | 14 | 15 | | 75 | | 16 | | 0.755 ± 0.159 | | 0.018 | | (0.012, 0.029) | 0.1 | (0.072, 0.139) | 15.991 |
| JeL | 14 | 15 | | 55 | | 23 | | 1.333 ± 0.108 | | 0.063 | | (0.058, 0.068) | 0.165 | (0.139, 0.194) | 38.408 |
| JeL | 14 | 20 | | 75 | | 20 | | 1.703 ± 0.128 | | 0.09 | | (0.084, 0.096) | 0.19 | (0.164, 0.221) | 68.586 |
| JeL | 14 | 20 | | 55 | | 18 | | 1.755 ± 0.167 | | 0.052 | | (0.049, 0.055) | 0.108 | (0.093, 0.125) | 15.038 |
| JeL | 14 | 25 | | 75 | | 17 | | 1.396 ± 0.17 | | 0.046 | | (0.042, 0.05) | 0.116 | (0.094, 0.142) | 21.359 |
| JeL | 14 | 25 | | 55 | | 15 | | 0.645 ± 0.202 | | 0.015 | | (0.007, 0.032) | 0.113 | (0.065, 0.196) | 16.543 |
| JeL | 14 | 30 | | 75 | | 18 | | 0.935 ± 0.134 | | 0.09 | | (0.079, 0.102) | 0.353 | (0.248, 0.505) | 77.731 |
| JeL | 14 | 30 | | 55 | | 10 | | 0.323 ± 0.158 | | 0.004 | | (0, 0.044) | 0.21 | (0.046, 0.963) | 38.129 |
| JeL | 21 | 15 | | 75 | | 14 | | 0.681 ± 0.194 | | 0.014 | | (0.007, 0.029) | 0.094 | (0.061, 0.144) | 11.935 |
| JeL | 21 | 15 | | 55 | | 18 | | 1.791 ± 0.166 | | 0.047 | | (0.044, 0.05) | 0.097 | (0.085, 0.11) | 22.255 |
| JeL | 21 | 20 | | 75 | | 18 | | 1.228 ± 0.148 | | 0.08 | | (0.071, 0.089) | 0.226 | (0.164, 0.312) | 38.951 |
| JeL | 21 | 20 | | 55 | | 16 | | 1.657 ± 0.197 | | 0.037 | | (0.034, 0.041) | 0.081 | (0.07, 0.093) | 21.244 |
| JeL | 21 | 25 | | 75 | | 17 | | 1.495 ± 0.18 | | 0.039 | | (0.035, 0.043) | 0.091 | (0.077, 0.107) | 14.212 |
| JeL | 21 | 25 | | 55 | | 8 | | 1.495 ± 0.18 | | NA | | (NA, NA) | NA | (NA, NA) | 7.5 |

Table 5: List of treatment combinations where significant differences were determined by comparison of the confidence intervals for LC_50_.

| **After 7 days** | | | | |  | **After 14 days** | | | | |  | **After 21 days** | | | | |
| --- | --- | --- | --- | --- | --- | --- | --- | --- | --- | --- | --- | --- | --- | --- | --- | --- |
|  | **Comparisons** | | | |  |  | **Comparisons** | | | |  |  | **Comparisons** | | | |
| Treat | Temp | | RH | |  | Treat | Temp | | RH | |  | Treat | Temp | | RH | |
| SmL | 15 | 20 | 75 | 55 |  | SmL | 15 | 20 | 55 | 55 |  | SmL | 15 | 20 | 55 | 55 |
| SmL | 15 | 20 | 75 | 75 |  | SmL | 15 | 20 | 55 | 75 |  | SmL | 15 | 20 | 55 | 75 |
| SmL | 15 | 25 | 75 | 55 |  | SmL | 15 | 25 | 55 | 75 |  | SmL | 15 | 25 | 55 | 55 |
| SmL | 15 | 25 | 75 | 75 |  | SmL | 15 | 30 | 55 | 55 |  | SmL | 15 | 25 | 55 | 75 |
| SmL | 15 | 30 | 75 | 55 |  | SmL | 15 | 20 | 75 | 55 |  | SmL | 15 | 30 | 55 | 55 |
| SmL | 15 | 30 | 75 | 75 |  | SmL | 15 | 20 | 75 | 75 |  | SmL | 15 | 20 | 75 | 55 |
| SmL | 20 | 25 | 55 | 75 |  | SmL | 15 | 25 | 75 | 75 |  | SmL | 15 | 20 | 75 | 75 |
| SmL | 20 | 30 | 55 | 75 |  | SmL | 15 | 30 | 75 | 55 |  | SmL | 15 | 25 | 75 | 55 |
| SmL | 20 | 25 | 75 | 75 |  | SmL | 15 | 30 | 75 | 75 |  | SmL | 15 | 25 | 75 | 75 |
| SmL | 20 | 30 | 75 | 75 |  | SmL | 20 | 20 | 55 | 75 |  | SmL | 15 | 30 | 75 | 55 |
| SmL | 25 | 25 | 55 | 75 |  | SmL | 20 | 25 | 55 | 55 |  | SmL | 20 | 20 | 55 | 75 |
| SmL | 25 | 30 | 55 | 75 |  | SmL | 20 | 30 | 55 | 75 |  | SmL | 20 | 30 | 55 | 75 |
| SmL | 25 | 30 | 75 | 55 |  | SmL | 20 | 25 | 75 | 75 |  | SmL | 20 | 25 | 75 | 55 |
| SmL | 25 | 30 | 75 | 75 |  | SmL | 20 | 30 | 75 | 55 |  | SmL | 20 | 25 | 75 | 75 |
| SmL | 30 | 30 | 55 | 75 |  | SmL | 25 | 25 | 55 | 75 |  | SmL | 20 | 30 | 75 | 55 |
| SmZ | 15 | 15 | 55 | 75 |  | SmL | 25 | 30 | 55 | 55 |  | SmL | 20 | 30 | 75 | 75 |
| SmZ | 15 | 20 | 55 | 75 |  | SmL | 25 | 30 | 75 | 55 |  | SmL | 25 | 30 | 55 | 75 |
| SmZ | 15 | 25 | 55 | 55 |  | SmL | 25 | 30 | 75 | 75 |  | SmL | 25 | 30 | 75 | 75 |
| SmZ | 15 | 25 | 55 | 75 |  | SmL | 30 | 30 | 55 | 75 |  | SmL | 30 | 30 | 55 | 75 |
| SmZ | 15 | 30 | 55 | 55 |  | SmZ | 15 | 15 | 55 | 75 |  | SmZ | 15 | 15 | 55 | 75 |
| SmZ | 15 | 25 | 75 | 75 |  | SmZ | 15 | 20 | 55 | 55 |  | SmZ | 15 | 20 | 55 | 55 |
| SmZ | 15 | 30 | 75 | 55 |  | SmZ | 15 | 20 | 55 | 75 |  | SmZ | 15 | 25 | 55 | 55 |
| SmZ | 15 | 30 | 75 | 75 |  | SmZ | 15 | 25 | 55 | 55 |  | SmZ | 15 | 25 | 55 | 75 |
| SmZ | 20 | 20 | 55 | 75 |  | SmZ | 15 | 25 | 55 | 75 |  | SmZ | 15 | 30 | 55 | 55 |
| SmZ | 20 | 25 | 55 | 75 |  | SmZ | 15 | 30 | 55 | 55 |  | SmZ | 15 | 20 | 75 | 75 |
| SmZ | 20 | 30 | 55 | 55 |  | SmZ | 15 | 20 | 75 | 55 |  | SmZ | 15 | 25 | 75 | 75 |
| SmZ | 20 | 25 | 75 | 75 |  | SmZ | 15 | 20 | 75 | 75 |  | SmZ | 15 | 30 | 75 | 75 |
| SmZ | 20 | 30 | 75 | 55 |  | SmZ | 15 | 25 | 75 | 55 |  | SmZ | 20 | 20 | 55 | 75 |
| SmZ | 20 | 30 | 75 | 75 |  | SmZ | 15 | 25 | 75 | 75 |  | SmZ | 20 | 25 | 55 | 75 |
| SmZ | 25 | 25 | 55 | 75 |  | SmZ | 15 | 30 | 75 | 75 |  | SmZ | 20 | 30 | 55 | 75 |
| SmZ | 25 | 30 | 55 | 55 |  | SmZ | 20 | 20 | 55 | 75 |  | SmZ | 20 | 25 | 75 | 55 |
| SmZ | 25 | 30 | 55 | 75 |  | SmZ | 20 | 25 | 55 | 75 |  | SmZ | 20 | 25 | 75 | 75 |
| SmZ | 25 | 30 | 75 | 55 |  | SmZ | 20 | 30 | 55 | 55 |  | SmZ | 20 | 30 | 75 | 55 |
| SmZ | 25 | 30 | 75 | 75 |  | SmZ | 20 | 30 | 55 | 75 |  | SmZ | 25 | 30 | 55 | 75 |
| SmZ | 30 | 30 | 55 | 75 |  | SmZ | 20 | 25 | 75 | 55 |  | SmZ | 25 | 30 | 75 | 55 |
| JeL | 15 | 15 | 55 | 75 |  | SmZ | 20 | 25 | 75 | 75 |  | SmZ | 25 | 30 | 75 | 75 |
| JeL | 15 | 20 | 55 | 55 |  | SmZ | 20 | 30 | 75 | 55 |  | SmZ | 30 | 30 | 55 | 75 |
| JeL | 15 | 20 | 55 | 75 |  | SmZ | 20 | 30 | 75 | 75 |  | JeL | 15 | 15 | 55 | 75 |
| JeL | 15 | 25 | 55 | 55 |  | SmZ | 25 | 25 | 55 | 75 |  | JeL | 15 | 20 | 55 | 55 |
| **After 7 days** | | | | |  | **After 14 days** | | | | |  | **After 21 days** | | | | |
|  | **Comparisons** | | | |  |  | **Comparisons** | | | |  |  | **Comparisons** | | | |
| Treat | Temp | | RH | |  | Treat | Temp | | RH | |  | Treat | Temp | | RH | |
| JeL | 15 | 25 | 55 | 75 |  | SmZ | 25 | 30 | 55 | 55 |  | JeL | 15 | 20 | 55 | 75 |
| JeL | 15 | 30 | 55 | 55 |  | SmZ | 25 | 30 | 55 | 75 |  | JeL | 15 | 25 | 55 | 75 |
| JeL | 15 | 30 | 55 | 75 |  | SmZ | 25 | 30 | 75 | 55 |  | JeL | 15 | 30 | 55 | 75 |
| JeL | 15 | 20 | 75 | 55 |  | SmZ | 25 | 30 | 75 | 75 |  | JeL | 15 | 20 | 75 | 55 |
| JeL | 15 | 25 | 75 | 55 |  | SmZ | 30 | 30 | 55 | 75 |  | JeL | 15 | 20 | 75 | 75 |
| JeL | 15 | 25 | 75 | 75 |  | JeL | 15 | 15 | 55 | 75 |  | JeL | 15 | 25 | 75 | 75 |
| JeL | 15 | 30 | 75 | 55 |  | JeL | 15 | 20 | 55 | 55 |  | JeL | 15 | 30 | 75 | 75 |
| JeL | 15 | 30 | 75 | 75 |  | JeL | 15 | 20 | 55 | 75 |  | JeL | 20 | 20 | 55 | 75 |
| JeL | 20 | 20 | 55 | 75 |  | JeL | 15 | 25 | 55 | 55 |  | JeL | 20 | 30 | 55 | 75 |
| JeL | 20 | 25 | 55 | 55 |  | JeL | 15 | 25 | 55 | 75 |  | JeL | 20 | 25 | 75 | 75 |
| JeL | 20 | 25 | 55 | 75 |  | JeL | 15 | 30 | 55 | 55 |  | JeL | 25 | 30 | 75 | 75 |
| JeL | 20 | 30 | 55 | 55 |  | JeL | 15 | 30 | 55 | 75 |  |  |  |  |  |  |
| JeL | 20 | 30 | 55 | 75 |  | JeL | 15 | 20 | 75 | 55 |  |  |  |  |  |  |
| JeL | 20 | 25 | 75 | 55 |  | JeL | 15 | 20 | 75 | 75 |  |  |  |  |  |  |
| JeL | 20 | 25 | 75 | 75 |  | JeL | 15 | 25 | 75 | 75 |  |  |  |  |  |  |
| JeL | 20 | 30 | 75 | 55 |  | JeL | 15 | 30 | 75 | 75 |  |  |  |  |  |  |
| JeL | 20 | 30 | 75 | 75 |  | JeL | 20 | 20 | 55 | 75 |  |  |  |  |  |  |
| JeL | 25 | 25 | 55 | 75 |  | JeL | 20 | 25 | 55 | 55 |  |  |  |  |  |  |
| JeL | 25 | 30 | 55 | 55 |  | JeL | 20 | 30 | 55 | 55 |  |  |  |  |  |  |
| JeL | 25 | 30 | 55 | 75 |  | JeL | 20 | 30 | 55 | 75 |  |  |  |  |  |  |
| JeL | 25 | 30 | 75 | 55 |  | JeL | 20 | 25 | 75 | 55 |  |  |  |  |  |  |
| JeL | 25 | 30 | 75 | 75 |  | JeL | 20 | 25 | 75 | 75 |  |  |  |  |  |  |
| JeL | 30 | 30 | 55 | 75 |  | JeL | 20 | 30 | 75 | 55 |  |  |  |  |  |  |
| SiS | 15 | 20 | 55 | 75 |  | JeL | 25 | 25 | 55 | 75 |  |  |  |  |  |  |
| SiS | 15 | 25 | 55 | 75 |  | JeL | 25 | 30 | 55 | 75 |  |  |  |  |  |  |
| SiS | 15 | 30 | 55 | 55 |  | JeL | 25 | 30 | 75 | 75 |  |  |  |  |  |  |
| SiS | 20 | 25 | 75 | 75 |  | JeL | 30 | 30 | 55 | 75 |  |  |  |  |  |  |
| SiS | 20 | 30 | 75 | 75 |  |  |  |  |  |  |  |  |  |  |  |  |
| SiS | 25 | 30 | 75 | 55 |  |  |  |  |  |  |  |  |  |  |  |  |
| SiS | 25 | 30 | 75 | 75 |  |  |  |  |  |  |  |  |  |  |  |  |
| SiS | 30 | 30 | 55 | 75 |  |  |  |  |  |  |  |  |  |  |  |  |
